# ImmTree: Database of evolutionary relationships of genes and proteins in the human immune system

**DOI:** 10.1186/1745-7580-3-4

**Published:** 2007-03-21

**Authors:** Csaba Ortutay, Markku Siermala, Mauno Vihinen

**Affiliations:** 1 Institute of Medical Technology, FI-33014 University of Tampere, Finland; 2 Research Unit, Tampere University Hospital, FI-33520 Tampere, Finland

## Abstract

**Background:**

The immune system, which is a complex machinery, is based on the highly coordinated expression of a wide array of genes and proteins. The evolutionary history of the human immune system is not well characterised. Although several studies related to the development and evolution of immunological processes have been published, a full-scale genome-based analysis is still missing. A database focused on the evolutionary relationships of immune related genes would contribute to and facilitate research on immunology and evolutionary biology.

**Results:**

An Internet resource called ImmTree  was constructed for studying the evolution and evolutionary trees of the human immune system. ImmTree contains information about orthologs in 80 species collected from the HomoloGene, OrthoMCL and EGO databases. In addition to phylogenetic trees, the service provides data for the comparison of human-mouse ortholog pairs, including synonymous and non-synonymous mutation rates, Z values, and K_a_/K_s _quotients. A versatile search engine allows complex queries from the database. Currently, data is available for 847 human immune system related genes and proteins.

**Conclusion:**

ImmTree provides a unique data set of genes and proteins from the human immune system, their phylogenetics, and information for comparisons of human-mouse ortholog pairs, synonymous and non-synonymous mutation rates, as well as other statistical information.

## Background

The immune system is a very complex machinery thet has evolved and diversified over time. Numerous processes are necessary for mounting adaptive and innate immune responses to protect an individual from invading organisms and molecules. Acquired and congenital problems in almost any part of the immune system can lead to diseases, many of which are very severe or even life threatening. The different processes and pathways of the immune system have evolved gradually and become increasingly complex. More ancient innate or intrinsic immunity has been further complemented by adaptive processes, which provide a specific response when required.

Although intensively studied, the evolutionary history of this system is not well known. The evolution of certain immunological protein groups of the human immunome have already been studied. For example, five gene groups of the NF-κB signaling pathway in vertebrates and insects [1], or the evolution of the interleukin-1 protein family in vertebrates [2] are extensively studied. To explore the molecular evolution of the human immune system, a reference set of genes and proteins needs to be defined [3]. We have identified and collected genes and proteins essential for human immunity and a genome wide investigation of the evolution of these genes has been carried out [4]. Here, we describe a database for the evolutionary trees of proteins in the human immune system (ImmTree) [5]. ImmTree contains information for orthologs of the human genes in 80 species, including all the major model organisms from Eukaryota. The evolutionary relationships of the orthologs are presented as phylogenetic trees. Further, ImmTree provides a unique data set for comparison of human-mouse ortholog pairs by the presented synonymous and non-synonymous mutation rates of the genes.

## Construction and content

### Collecting human immune system related genes and proteins and their orthologs

We collected from articles, textbooks and electronic sources altogether 847 human genes that are involved in immunology related processes, or which are essential for the life of immunological cells and organs [4]. The variable chains of the immunoglobulins (Igs), B and T cell receptors (BCRs and TCRs) and major histocompatibility complexes (MHCs) were not included since these proteins are not coded by conventionally structured genes but by gene fragments. These gene fargments and their products are already exclusively collected and listed in IMGT, the international ImMunoGeneTics information system at National Computer Centre of Higher Education [6] and European Bioinformatics Institute [7]. ImmTree contains the genes and proteins that are required for processing these gene fragments. In the ImmTree database Entrez Gene [8] identifiers were used to refer to genes. Protein sequences were downloaded from NCBI GenBank [9]. Ortholog sequences are from the Eukaryotic Gene Orthologs (EGO) [10], HomoloGene [11] and OrthoMCL [12] databases. HomoloGene contains groups of homologs for completely sequenced eukaryotic genomes, while EGO has (tentative) ortholog groups of the eukaryotic sequences in the TIGR sequence database. OrthoMCL contains sequences exclusively from 55 complete genomes and therefore the number of sequences from the different branches is limited. The releases used were EGO version 9.0, released 15 February 2005; HomoloGene build 50.1, released 25 July 2006; Ortho MCL version 1.0, released 19 October 2005.

The nucleotide sequences of ortholog groups were taken from EGO and the protein sequences from HomoloGene and OrthoMCL. The sequences were aligned using ClustalW [13] with the default parameters. Phylogenetic trees were reconstructed for all three type of ortholog groups using the PAUP* program package [14] when the group contained at least three sequences. We thus created three trees for most of the ortholog groups for the data from the three independent databases. A simple neighbour-joining method was applied if the ortholog group contained only three taxa, otherwise bootstrap analysis was applied with parsimony method, heuristic tree search, and 1000 replications. The number of bootstrap replicates was reduced to 100 in the case of OrthoMCL ortholog groups where more than 50 sequences were in the group. Similarly the number of replicates was reduced even further, to 50, where the number of sequences exceeded 100. This was necessary due to computational time requirements, since some OrthoMCL groups contain numerous paralogs. In these cases, the tree constructing becomes very CPU intensive without any further phylogenetic advantage.

For a general overview of the ortholog groups, we generated a fourth tree. This tree represents protein sequences from all the species in any of the three datasets. Moreover, each species is represented by just one sequence, preventing the accumulation of identical sequences from multiple data sources. This way the large paralog groups from the OrthoMCL database are represented by just a single sequence.

The nucleotide sequences from the EGO database were translated to amino acids to align the representative protein sequences from the three databases. The translation was done in all six frames, and all six transcripts were aligned with the human protein sequence using bl2seq from the BLAST package [15]. Only the transcript with the longest identical stretch with the human ortholog was retained for further analysis. The protein sequences collected this way were aligned and phylogenetic trees were constructed as described above.

### Comparison of the human-mouse ortholog pairs

In 603 cases orthologs were present both in the mouse and human genome in the HomoloGene database. These pairs were further analysed in detail. The cDNA sequences of the human and mouse genes were translated to protein sequences and then aligned using the blast2seq program. The corresponding cDNA sequences were aligned based on the amino acid sequence alignment with proprietary Perl scripts, some of which utilize modules from the Bioperl Project [16]. The estimates of synonymous mutations per synonymous sites (K_s _or dS) and of non-synonymous mutations per non-synonymous sites (K_a _or dN) values were calculated [17]. Z values and the K_a_/K_s _quotients describe the conservation of given genes since the human-mouse divergence.

## Utility and Discussion

### Database access and search

**Figure 1:**
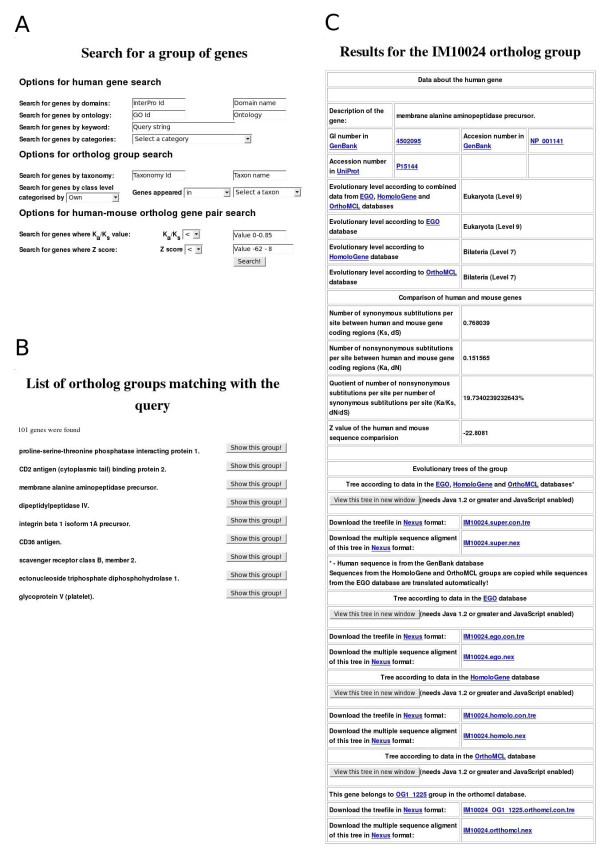
**Examples of ImmTree search functions and data presentation**. A) Search form for a gene group. B) Top of the result list for query 'genes which appeared earlier than group Coelomata in the EGO database' C) Information in ImmTree for the human membrane alanine aminopeptidase precursor and its orthologs.

The ImmTree database can be accessed online [5]. The service provides two search modes. The first search page is an interface for finding human genes by GenBank gi numbers, GenBank accession numbers, or UniProt [18] accession numbers. The other engine is for searching ortholog groups by using more complex criteria (Fig 1A). The first options concentrate on features of human genes and proteins. One can search for protein domains either by InterPro [19] id or name of the domain. Ontology queries are based either on GeneOntology [20] ids or ontology keywords. In addition, keyword searches are possible for gene identification. Also, some predefined categories like 'CD molecules', 'complement system' or 'inflammation' can be searched.

The second group of search options helps to identify features common for ortholog groups. The most basic option is to search for organisms within an ortholog group either by NCBI's Taxonomy [21] id or by the name of the taxon. Ortholog groups can be searched also by their ancestor taxa. Such complex searches can be performed, for example, only for 'genes which appeared earlier than Coelomata according to the EGO database' (94 result groups) or 'genes which emerged in the Bilateria group according to the HomoloGene database' (41 result groups).

The third type of search option is based on the statistical information of human-mouse ortholog pair comparisons. Gene pairs can be found by the K_a_/K_s _quotient value or by the Z value. Both these parameters refer to the conservation of sequences [22,23]. Is is also possible to combine search options, for example, to search for the 'genes related to the complement system which have a K_a_/K_s _value less than 0.15' (5 result groups) or 'genes with the keyword lectin which have a K_a_/K_s _value greater than 0.6' (4 result groups).

### Reports of results

All the search results are displayed in an interactive list from which one can investigate details for each of the identified ortholog groups (Fig 1B). Similarly to the gene group search page, the results for a single ortholog group are divided into three main parts (Fig 1C). The header of the page presents details of the human gene. Sequences are available via links to GenBank and UniProt. Evolutionary levels denoting the appearance of the gene are shown based on the EGO, HomoloGene and OrthoMCL databases and combined data. Then, the results of the human-mouse ortholog comparison, including the values for the number of synonymous and nonsynonymous substitutions per site (K_s_, K_a_), their quotient value (K_a_/K_s_) and Z value, are presented. The evolutionary trees for the combined, EGO, HomoloGene and OrthoMCL datasets are in the third section. Links for the trees for the four datasets are also provided. The multiple sequence alignments and the evolutionary trees are available in nexus format [24] for download and can be visualized with the ATV (A Tree Viewer) Java Applet [25].

**Figure 2:**
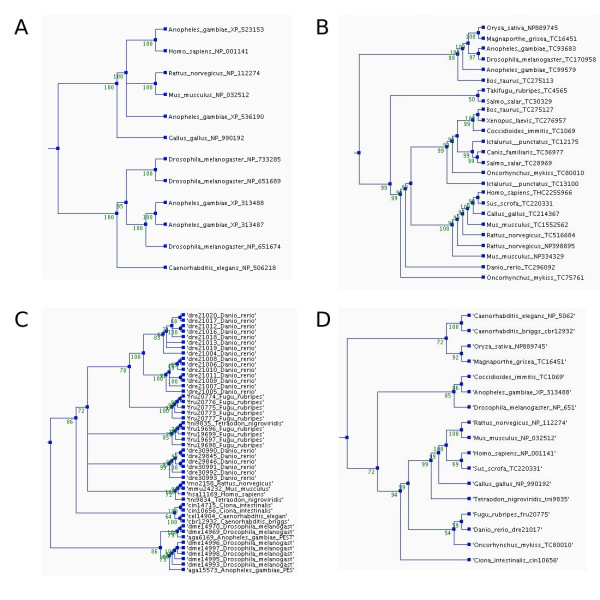
**Phylogenetic trees for the orthologs of the human membrane alanine aminopeptidase precursor in the different ortholog databases**. The trees were constructed using parsimony method, a heuristic tree search, and 1000 replications. Bootstrap values are shown at the nodes. The trees and multiple sequence alignments can be downloaded from the ImmTree database. A) Orthologs in HomoloGene, B) EGO, and C) OrthoMCL (Note the number of paralogs.) D) Overview tree presenting one sequence for each species in any of the databases.

Figure 2 presents the four phylogenetic trees for the orthologs of the human membrane alanine aminopeptidase precursor. The differences of the ortholog definitions in the different databases are clearly visible. The most strict definition of an ortholog group is in the HomoloGene database (Fig 2A). There are sequences just from a few species, and just a few paralogs in the dataset. Contrastingly, the tree for the EGO data (Fig 2B) contains sequences from more species. EGO's definition of an ortholog group is less strict and therefore the groups are called tentative ortholog groups. Consequently the sequences are usually more distant. Many OrthoMCL groups (Fig 2C) contain lots of paralogs. OrthoMCL includes proteins from only 55 selected genomes. Paralogs are presented if they appeared after the most recent divergence of the included genomes. EGO and HomoloGene have sequences from a much broader species spectrum, and in addition they try to avoid the inclusion of paralogs. In ImmTree all three datasets with the corresponding trees are provided, and the user can use any of them according to their needs. For a more general overview, ImmTree provides a fourth tree (Fig 2D) to combine the data from the three databases. In this tree, only one sequence from each species is included. ImmTree thus allows one to investigate how broadly spread genes are among the taxa.

## Conclusion

ImmTree is a new and unique data resource for exploring the molecular evolution of the immune system. Although excellent databases, such as The Adaptive Evolution Database (TAED) [26] or the Database of Evolutionary Distances (DED) [27] are available for studying molecular evolution, they are general systems for all genes. It would be hard to collect molecular evolution related data for the immune system from them. ImmTree is a dedicated resource considering the special needs of researchers of evolution of the immune system. ImmTree facilitates queries according to the classic groupings of immune functions, such as humoral immunity, cellular immunity, complement system. The database will be continuously updated.

## Availability and requirements

The ImmTree database is freely available for academic use from the URL: 

## Competing interests

The author(s) declare that they have no competing interests.

## Authors' contributions

CO and MS collected the sequences of the immunome genes. CO carried out the phylogenetic analysis and MS collected the identification numbers connected to the immunome genes. MV designed and coordinated the project and compiled the list of genes and proteins. All authors drafted the manuscript and approved its content.
